# Regulation of miRNAs Expression by Mutant p53 Gain of Function in Cancer

**DOI:** 10.3389/fcell.2021.695723

**Published:** 2021-12-06

**Authors:** Tzitzijanik Madrigal, Jesús Hernández-Monge, Luis A Herrera, Claudia Haydée González-De la Rosa, Guadalupe Domínguez-Gómez, Myrna Candelaria, Fernando Luna-Maldonado, Karla G Calderón González, José Díaz-Chávez

**Affiliations:** ^1^ Unidad de Investigación Biomédica en Cáncer, Instituto de Investigaciones Biomédicas, UNAM/Instituto Nacional de Cancerología, Mexico City, Mexico; ^2^ Departamento de Ciencias Biológicas y de La Salud, UAM Iztapalapa, Mexico City, Mexico; ^3^ Cátedra-CONACyT Laboratorio de Biomarcadores Moleculares, Instituto de Física, UASLP, San Luis Potosí, Mexico; ^4^ Instituto Nacional de Medicina Genómica, Mexico City, Mexico; ^5^ Departamento de Ciencias Naturales, Unidad Cuajimalpa, Universidad Autónoma Metropolitana, Mexico City, Mexico; ^6^ Subdirección de Investigación Clínica, Instituto Nacional de Cancerología, Mexico City, Mexico; ^7^ Laboratorio de Interacciones Biomoleculares y Cáncer, Instituto de Física, UASLP, San Luis Potosi, Mexico

**Keywords:** mutant p53, miRNAs, cancer, gain of function, miRNA biogenesis

## Abstract

The p53 roles have been largely described; among them, cell proliferation and apoptosis control are some of the best studied and understood. Interestingly, the mutations on the six hotspot sites within the region that encodes the DNA-binding domain of p53 give rise to other very different variants. The particular behavior of these variants led to consider p53 mutants as separate oncogene entities; that is, they do not retain wild type functions but acquire new ones, namely Gain-of-function p53 mutants. Furthermore, recent studies have revealed how p53 mutants regulate gene expression and exert oncogenic effects by unbalancing specific microRNAs (miRNAs) levels that provoke epithelial-mesenchymal transition, chemoresistance, and cell survival, among others. In this review, we discuss recent evidence of the crosstalk between miRNAs and mutants of p53, as well as the consequent cellular processes dysregulated.

## Introduction

**FIGURE 1:**
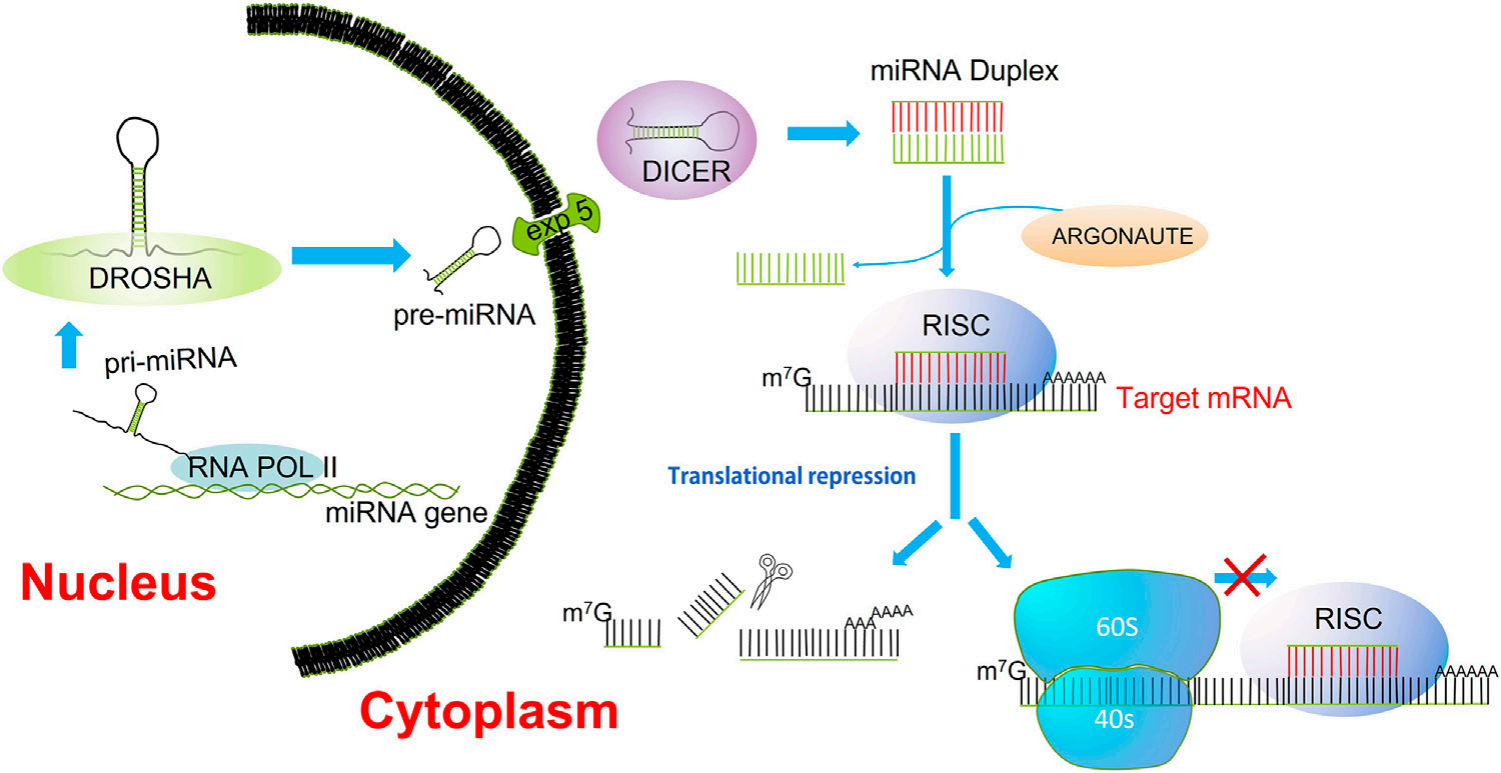
miRNA biogenesis. Transcription of miRNAs is mediated by RNA polymerase II. The pri-miRNAs are substrates for the rnase III, Drosha. The product of pre-miRNA cleavage by Drosha is exported to the cytoplasm by the nuclear transporter exportin 5. In the cytoplasm, DICER processes the pre-miRNA into a miRNA/miRNA* duplex. Final processing yields the mature miRNA duplex, the strand with lower thermodynamic stability is loaded into Argonaute (AGO); this is deemed the guide strand. The guide strand alongside AGO constitutes the minimal RNA-induced silencing complex (RISC). Once recruited the RISC complex onto the mRNA target, the inhibition of translation is carried out through cleavage, deadenylation, or blocking of the mRNA translation.

The miRNAs are short non-coding RNAs that function as post-transcriptional regulators of gene expression. (Jansson and Lund, 2012). Many miRNAs are found evolutionarily conserved in several organisms, which are suggested to have an important function in the essential biological processes (Jones and Lal, 2012). The miRNA biogenesis starts with the transcription of pri-miRNAs by RNA polymerase II from introns or exons of host genes but also from their promoters. The pri-miRNA forms a hairpin structure that is recognized and processed by the RNA binding protein Di George Syndrome (DGCR8) and a ribonuclease III enzyme (Drosha); the resulting product, a pre-miRNA is exported to the cytoplasm by exportin 5/RAN GTP complex. Within the cytoplasm, the pre-miRNA is cleaved by the rnase III endonuclease Dicer, giving rise to a mature miRNA duplex (Denli et al., 2004). The Argonaute family of proteins (AGO1-4) can be loaded with any strand of the miRNA duplex, but the strand with lower stability is more likely loaded into AGO (Ha and Kim, 2014). The complex formed by the guide strand and AGO is considered the minimal miRNA-induced silencing complex (miRISC). A perfect pairing between miRNA and its mRNA target induces rapid Poly(a)-deadenylation and decapping steps, which cleavage and degrade the mRNA target. However, most interactions are not fully complementary; therefore, the miRISC complex in the first place interferes with the translation initiation (Li et al., 2014a; O'Brien et al., 2018) (Figure 1). Moreover, a given mRNA can be concurrently regulated by multiple miRNAs, and an estimated 60% of the human genome is regulated by miRNAs (Hermeking, 2012).

miRNAs are regulators of numerous cellular processes, including proliferation, differentiation, and apoptosis (Hainaut and Hollstein, 2000; Hermeking, 2012). Many miRNAs map to specific regions of the human genome frequently deleted or enhanced in human cancers (Croce, 2009). Growing evidence demonstrates that they form unique expression patterns or signatures (Calin and Croce, 2006; Donzelli et al., 2014). Interestingly, it has been reported that p53, the most frequently mutated gene in human cancer, modulates miRNA expression (Hermeking, 2012; Jones and Lal, 2012; Li et al., 2014a). The mutations in the gene *TP53* are mainly missense, resulting in the production of a full-length mutant p53 protein, unlike most tumor suppressor genes inactivated through biallelic deletion or truncation mutations (Li et al., 2014a; Muller and Vousden, 2014). The mutations of p53 are frequently in six ‘hotspot’ residues within the DNA-binding domain: R175, G245, R248, R249, R273, and R282 (Hainaut and Hollstein, 2000; Mello and Attardi, 2013). Because these missense mutations are mainly located in the DNA-binding domain of p53, the mutant p53 protein is unable to transactivate most of its target genes resulting in a loss of the protein p53 wild type function. Besides, the mutant p53 protein can, in many cases, lose its functions and may exert a dominant negative regulation on any remaining p53 wt (Petitjean et al., 2007).

Moreover, mutant p53 also acquires oncogenic functions that modulate various phenotypes such as epithelial-mesenchymal transition (EMT), migration, invasion, metastasis, chemoresistance, proliferation, apoptosis and genomic instability (Li et al., 2014a); these functions are entirely independent of p53 wt function (Strano et al., 2007; Brosh and Rotter, 2009). The mutations of p53 can be divided into two main classes: 1) DNA contact defective mutants, whose residue subjected to mutation is located in the region of the protein that binds to DNA (R273H, R273C, R248Q, and R248W) and 2) Structural defective mutants, whose mutation impairs a residue critical for the entire folding of the protein (R175H, G245S, R249S, and R282H) (Bullock and Fersht, 2001). In addition, recent studies have shown that mutant p53 can regulate gene expression and exert oncogenic effects through specific miRNAs (Li et al., 2014a).

Here, we review the mechanisms by which mutant p53 gains diverse oncogenic functions by regulating specific miRNAs.

## REGULATION of Diverse Cellular Processes Through miRNAs by MUTANT p53

### Cell Cycle and Apoptosis

#### miR-517a

The miR-517a fulfills physiological roles associated with the progression of pregnancy (Miura et al., 2010). It has also exhibited opposite functions regarding cellular proliferation or tumor suppressor activities. For instance, Yoshitomi and others observed that in bladder cancer cell lines, the overexpression of miR-517a led to inhibition of cell proliferation and increased cell apoptosis (Yoshitomi et al., 2011). Moreover, knockdown of miR-517a in glioma cells led to diminished cell proliferation and higher apoptosis; then, using a cell-line-derived xenograft mouse model, the U87 glioma cells lines expressing sh-miR-517a, showed lesser tumor growth as compared to the wild type cells (Du et al., 2019). Likewise, the silencing of miR-517a in melanoma cells induced up-regulation of CDKN1C (inhibitor of cyclin/cyclin-dependent kinase complexes), cleaved caspase-3, Bax/Bcl2 ratio, as well as high levels of Reactive Oxidative Species (ROS) and diminished cell proliferation (Yang et al., 2020). It is important to note that these reports do not clarify how miR-517a induces the aforementioned effects; meanwhile, other authors have been approaching identifying some of its targets. For instance, in SW48 and HCT116 colon cancer cells, an inverse correlation was identified between the expression of miR-517a and the forkhead box J3 (*FOXJ3*) tumor suppressor (Ma et al., 2016).

The expression of this and other microRNAs was assessed in the context of mutants of p53. Recently, Garibaldi and others carried out a genome wide expression analysis of 376 mature miRNA in SW480 mutant (p53R273H/P309S) colon cancer cells before and after constitutive depletion of the endogenous mutant p53. This analysis showed that mutant p53R273H downregulates 33 and upregulates four of 376 miRNAs (Garibaldi et al., 2016a). Among the data obtained, they observed that miR-520g, miR-518b, miR-582, miR-141, miR-519c, miR-143, miR-142-3p, and 142-5 were upregulated both at pri-miRNA and pre-miRNA levels after mutant p53 depletion, suggesting regulation at transcriptional level. Moreover, miR-517a, miR-519a, miR-105, miR-628, miR-1, miR-218, miR-515-3p, and miR-515-5p showed no significant change in primary transcripts after mutant p53 depletion, which was possibly due to a post-transcriptional regulation. After that, the authors demonstrated a post-transcriptional regulation of miRNAs through the interaction of mutp53 and the Drosha complex. This finding was corroborated by co-immunoprecipitation, confocal analysis, and RNA-chromatin immunoprecipitation experiments, which determined that endogenous mutant p53R273H directly binds to RNA helicases p72/82, hindering the association of this DEAD-box with other members of the microprocessor complex (Drosha), resulting in the inhibition of the miRNA biogenesis (Garibaldi et al., 2016a).

Although the overall biogenesis of miRNAs was hampered by mutant p53, one of the most downregulated miRNAs by the mutant p53R273H and p53R175H was miR-517a. In this regard, previous studies reported that miR-517a inhibits cell proliferation by blocking the G_2_/M transition in hepatocellular carcinoma cells (Liu et al., 2013). The underlying mechanism may involve the proline-rich tyrosine kinase 2 (Pyk2), which is a target of miR-517a; Pyk2 was shown to promote cell proliferation and invasiveness by upregulation of the c-Src and ERK/MAPK signaling pathways in hepatocellular carcinoma cells (Sun et al., 2008). Consistent with this, Garibaldi et al. demonstrated that the ectopic expression of the miR-517a impaired the SW480 cell survival as indicated by the reduction of viable cell number, the increase of trypan blue-positive cells, and by a diminished colony-forming ability, suggesting that this miRNA restrain the cell cycle progression. In addition, by cytofluorimetric assays, it was observed that miR-517a induced cell cycle arrest in G_2_/M, and induced apoptosis, which was determined by caspase activation, induction of cleaved PARP, and Annexin V assays (Garibaldi et al., 2016a).

**FIGURE 2:**
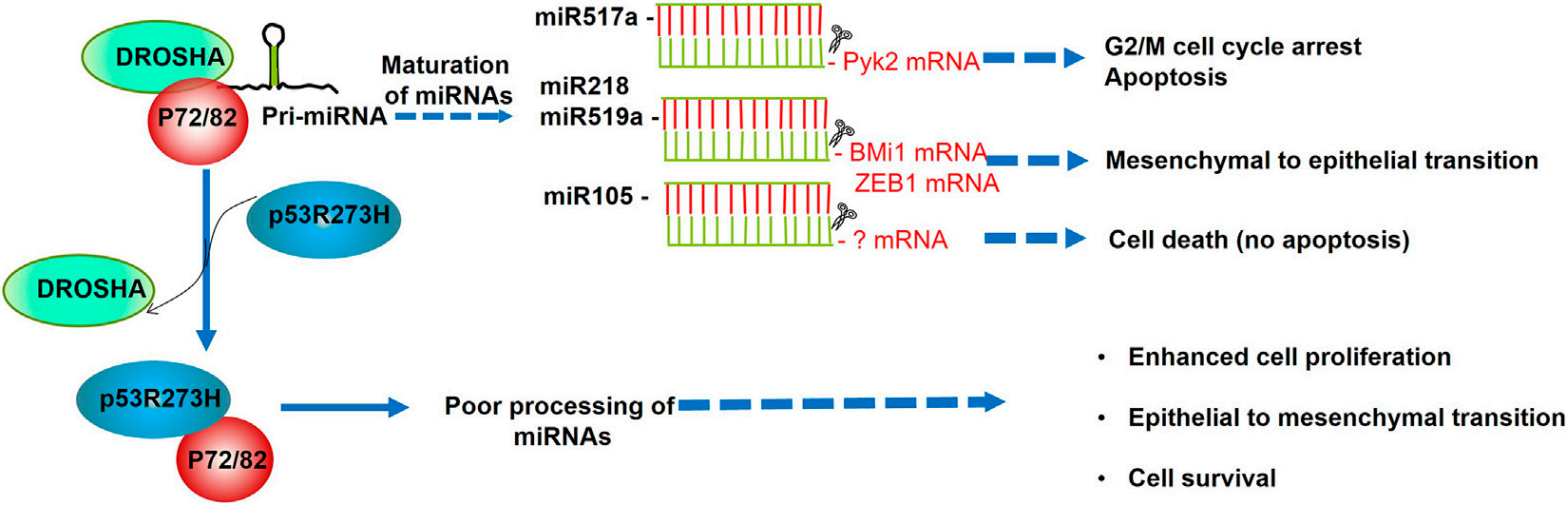
p53R273H promotes cell survival, proliferation, and epithelial to mesenchymal transition through sequestering p72/82. The association of p72/82, members of the DEAD-box family of RNA helicases, with DROSHA, is necessary to achieve a fine-tune processing of specific subsets of miRNAs. As p53R273H sequesters RNA helicases p72/82 from the microprocessor complex, this interferes with the maturation of miR517a, miR218, miR519a, and miR105. According to the biological processes regulated by these miRNAs, their downregulation provokes higher cell proliferation, cell survival, and EMT promotion.

Altogether, these functions of miR-517a pinpoint its important role as a tumor suppressor that is negatively regulated by the mutant p53R273H in colon cancer cells (Figure 2; Table 1).

**TABLE 1:** miRNAs Regulated by mutant p53.

*Mutant of p53*	*miRNA*	*Process*	*Reference*
p53R175H	miR-128-2	Cell cycle and apoptosis	Donzelli et al. (2012)
p53R175H	miR-223	Cell cycle and apoptosis	Masciarelli et al. (2014)
p53R273H	let-7i	Migration, Invasion and Metastasis	Subramanian et al. (2015)
p53R273H	miR-27a	Cell Proliferation	Wang et al. (2013)
p53G279E			
p53R175H			
p53R273H, p53R248Q, p53R175H*, p53C135Y*	miR-130b	Promote Epithelial Mesenchymal Transition	Dong et al. (2013)
p53R248Q, p53R282W*, p53R249S*	miR-155	Migration, Invasion, and Metastasis	Neilsen et al. (2013)
p53R273H*,* p53R175H	miR-517a	Cell cycle and apoptosis	Garibaldi et al. (2016a)
p53R273H	miR-519a	Promote Epithelial Mesenchymal Transition	Garibaldi et al. (2016a)
p53R273H	miR-105	Cell proliferation	Garibaldi et al. (2016a)
p53R273H	miR-218	Promote Epithelial Mesenchymal Transition	Garibaldi et al. (2016a)

#### miR-27a

miR-27a have a pivotal role in multiple processes, including cancer development, osteogenesis (Gu et al., 2016; You et al., 2016) adipogenesis (Kim et al., 2010; Hu et al., 2018), cell proliferation (Xu et al., 2013; Su et al., 2019), apoptosis (Sun et al., 2019) and differentiation (Kim et al., 2010; Tang et al., 2014).

**FIGURE 3:**
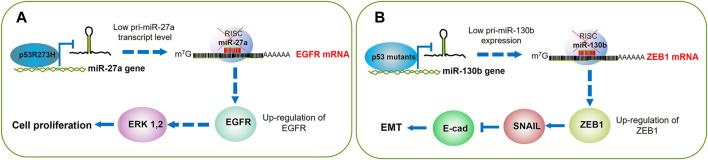
p53 mutants boost cell proliferation and EMT through gene promoter silencing. **(A)** p53R175H binds to and silences the miR-27a gene promoter; this leads to higher expression of its EGFR target. Then EGFR triggers the cell signaling that results in ERK 1,2 activation and cell cycle progression. **(B)** The mutants p53R175H, p53R273H, P53C135Y, and P53R248Q turn off miR130b expression, which results in the accumulation of ZEB1, that in turn activates SNAIL and consequent silencing of E-cadherin; altogether leading to activation of the EMT program.

Wang and others performed a custom miRNA microarray analysis to compare the miRNA expression profiles between p53 wt and mutant p53 in H1299 cells. The authors employed a p53-inducible system where the addition of doxycycline triggered the expression of p53 wt (H1299-Tet-On-p53) or mutant p53R273H (H1299-Tet-On-p53-273H). Mutant p53R273H exhibited differential expression of multiple miRNAs as compared with p53 wt. Among these findings, miR-27a was remarkably downregulated and furtherly studied. In addition to p53R273H, the mutants p53R175H and p53G279E, but not the wild-type p53, exhibited inhibitory effects on miR-27a expression. By chromatin immunoprecipitation (ChIP) assays, the authors determined a strong binding of mutant p53R273H with the miR-27a promoter within the nucleotide 2,899 to 2,675, indicating that this p53 mutant transcriptionally downregulates miR-27a. Next, the authors identified the epidermal growth factor receptor (*EGFR*) gene as a direct target of miR-27a; therefore, as p53R273H suppresses the expression of miR-27a, the *EGFR* expression increases, which leads to cell proliferation and tumor growth (Wang et al., 2013) (Figure 3A; Table 1). Concerning EGFR, some inhibitors that target this receptor’s kinase domain (TKIs) have been developed to tackle its permanent activation in patients with non-small cell lung cancer (Landi and Cappuzzo, 2013; Russo et al., 2017; Masood et al., 2019). Therefore, it would be interesting to analyze the response to TKIs among patients that harbor p53 mutations, especially those with lung cancer where the *EGFR* can be found overexpressed.

#### miR-105

miR-105 and other miRNAs exhibit opposite roles since they can behave like an oncogene or like a tumor suppressor. This miRNA was found upregulated in esophageal cancer tissues and was associated with positive lymph node metastasis, advanced TNM stage, and poor overall survival (Gao et al., 2020a). Conversely, miR-105 was downregulated in gastric cancer tissues, and its overexpression inhibited cell migration, invasion, and EMT in gastric cancer by targeting *SOX9* (Shang et al., 2019). In accordance, another report showed that up-regulation of miR-105 suppressed the colony formation and aggressiveness traits of gastric carcinoma cell lines BGC823 and SGC7901 *in vitro*. In this report, the authors also identified *SOX9* as the target of miR-105 (Jin et al., 2019). Since the role of miR-105 can apparently be interplayed as a tumor suppressor or as an oncogene, it would be difficult to bet for a certain gene therapy targeted to this microRNA.

Regarding mutant p53, the p53R273H impaired the expression of miR-105; the diminished level of this miRNA provoked high cell proliferation and low apoptosis. Conversely, when miR-105 was transiently transfected in SW480 cells, the cell proliferation did not change, but the cell death was evident (Garibaldi et al., 2016a). It is worth noting that the cell death was not apoptotic, and it remains to clarify what kind of cell death induces the miR-105 (Figure 2; Table 1).

It would be important to analyze whether the presence of other p53 mutations affects the expression of miR-105, also in other types of cancer, and whether it has or not a prognostic value.

### Chemoresistance and Cell Survival

#### miR-128-2

miR-128 is an intronic miRNA encoded by two different genes, miR-128-1 and miR-128-2, both located within the introns of *R3HDM1* and *ARPP21* genes, respectively (Li et al., 2013). It is considered that the function of miR-128 depends on the cellular context; however, it is mainly associated with the development and maintenance of the nervous system. Besides, it also has clear roles in the context of tumor cells as it was observed downregulated in glioblastoma, prostate cancer, lung cancer, colorectal, and breast cancer (Huang et al., 2015).

miR-128-3-p was used to enhance the chemosensitivity of colorectal cancer cells. This micro RNA was packed in a complex with PEG-PDMAEMA (Poly (ethylene glycol)–poly (2-(dimethylamino)ethyl methacrylate) that was following decorated with a peptide that targets the monocarboxylate transporter1 (tumor-homing peptide). This nanocomplex successfully delivered the micro RNA within cancer cells and inhibited the PI3K/AKT and MEK/ERK pathways. In combination with 5-Fluorouracil (5-FU), this nanocomplex dramatically improved the chemotherapy effects (Liu et al., 2020). In another interesting work, the authors used the intestinal Fetal Human Cells (FHC)for packing miR-128-3p within exosomes that were later exposed to oxaliplatin-resistant colorectal cancer cells. The authors observed a remarkable improvement in the oxaliplatin response and up-regulation of E-cadherin and reduced EMT (Liu et al., 2019).

**FIGURE 4:**
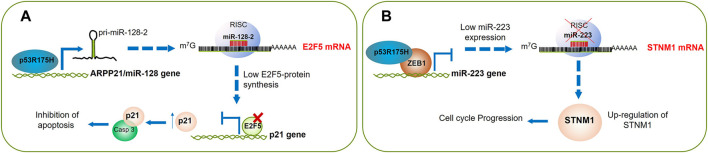
The cell cycle progression and inhibition of apoptosis can be triggered by p53R175H. **(A)** Mutant p53R175H induces miR-128-2 expression whose direct target is E2F5 mRNA. Downregulation of E2F5 blocks its repressive activity on p21 transcription, then p21 protein is accumulated in the cytoplasm, where it exerts an anti-apoptotic effect by binding to pro-caspase-3. **(B)** ZEB1 binds to miR-223 gene promoter and alongside p53R175H both inhibit miR-223 transcription, this allows increasing synthesis of STNM1 that is involved in chemoresistance and cell cycle progression.

Regarding p53 mutants, Donzelli and others searched for differential expression of a battery of miRNAs commonly altered in lung cancer in response to p53 mutants. Interestingly, using the H1299 cells, a human non-small-cell lung cancer (NSCLC) cell line, carrying a ponasterone (Pon-A) inducible mutant p53R175H protein; they found that the expression of intragenic miR-128-2 increases upon mutant p53R175H protein induction. Their studies demonstrated that the mutant p53 binds to the putative promoter of the miR-128-2 host gene, *ARPP21*, determining a concomitant induction of ARPP21 mRNA and miR-128-2 (Calin and Croce, 2006; Donzelli et al., 2012). To further investigate the contribution of miR-128-2 modulation to mutant p53 gain of function activity, they assessed whether miR-128-2 exogenous expression impacts the response of H1299 lung cancer cell line to doxorubicin (DOXO), cisplatinum (CDDP), and 5-fluorouracil (5-FU). They observed that miR-128-2 expression confers chemoresistance to all of these drugs. According to the experimental evidence, the authors further demonstrated that miR-128-2 post-transcriptionally targets E2F5, leading to the abrogation of its repressive activity on p21 transcription. Although nuclear p21 is strongly associated with growth arrest, it was observed that when p21 protein localizes to the cytoplasmic compartment, it exerts an anti-apoptotic effect by preventing pro-caspase three cleavage. Therefore, this study highlights the role of miRNA-128-2 on chemoresistance by inhibition of apoptosis in NSCLC (Donzelli et al., 2012) (Figure 4A; Table 1). Otherwise, considering that micro-RNAs potentially target more than one gene, it is quite possible that miR-128-2 may foster chemoresistance through the silencing of other genes different than *E2F5*. It would be also interesting to demonstrate whether other mutants of p53 can regulate the miR-128-2 expression; if this is the case, then it could also be a helpful marker to predict chemoresistance at least in NSCLC; yet, this effect is apparently context-dependent because, in colorectal cancer, glioblastoma, and ovarian cancer, the overexpression of miR-128-2 has been associated with chemosensitivity (Li et al., 2014b; She et al., 2014; Lian et al., 2018).

#### miR-223

miR-223 is located within the q12 locus of the X chromosome, and it is known to be an important factor for the differentiation and function of the immune system (Ye et al., 2018; Zhou et al., 2018). Regarding cancer, miR-223 has shown opposite roles because it has been observed upregulated in acute lymphoblastic leukemia and bladder cancer but downregulated in chronic lymphoid leukemia and hepatocellular carcinoma (Zhou et al., 2018). When miR-223 was overexpressed in HCT116 cells, the cell proliferation was increased, and the apoptosis diminished. These effects were presumably a consequence of the inhibition of the *FBXW7* tumor suppressor by miR-223 (Liu et al., 2021). Similarly, another study reported that miR-223 enhanced chemoresistance to cisplatin in human non-small cell lung cancer cells. The suggested underlying mechanism was the downregulation of *FBXW7* and subsequent activation of autophagy (Wang et al., 2020). In contrast, in breast cancer tumors, miR-223 was lost in the early stages, and its absence caused resistance to CDK4/6 inhibitors (Citron et al., 2020).

On the other hand, Donzelli (Donzelli et al., 2012), Masciarelli and others observed miR-223 downregulated upon expression of p53R175H (Masciarelli et al., 2014). To further investigate this finding in a more physiological context, they silenced the mutant p53 in SW480 (colon cancer cell line) or MDA-MB-468 and MDA-MB-231 cells (breast cancer cell lines), resulting in an increased miR-223 expression (Masciarelli et al., 2014). By chromatin immunoprecipitation (ChIP) assays, they provided evidence showing that p53 was bound to the miR-223 regulatory region. Interestingly, the authors also identified ZEB1 as an important transcription factor that cooperates with mutant p53 for the silencing of miR-223. Through ChIP-reChIP assays, they observed direct binding between mutant p53 and ZEB1 on the miR-223 regulatory region, concluding that mutant p53 does not bind directly to the DNA, but it does through ZEB1. The authors identified the miR-223 STMN1 target, a key microtubule-regulatory protein essential for cell cycle progression (Cimmino et al., 2005; Saito et al., 2015), as responsible for chemoresistance. *STMN1* was previously found downregulated by p53 wt (Ahn et al., 1999); it was associated with recurrence, metastasis, and drug resistance, and indeed, it has been studied as a therapeutic target (Rana et al., 2008; Phadke et al., 2011). In accordance, Masciarelli and others determined that silencing of *STMN1* in the mutant p53 SW480 cell line increased cell death in the presence of DNA damage induced by cisplatin. They also found that silencing of *STMN1* was associated with an inhibition of Cdk1 activity that provokes an arrest in Mitosis. Altogether, these data suggest that mutant p53R175H downregulated miR-223, which in turn upregulates *STMN1* expression leading to cancer cell resistance to chemotherapy in colon and breast cancer cell lines (Masciarelli et al., 2014). To further validate these findings, it will be important to determine if the presence of mutant p53R175H, p53R273H, or p53R280K and/or overexpression of *STMN1* are associated with chemoresistance in patients with colon and breast cancer; if this association is confirmed, it might be a valuable chemoresistance prognostic factor (Figure 4B; Table 1).

### Epithelial-Mesenchymal Transition (EMT)

#### miR-130b

miR-130b has been found either up or downregulated in several cancers; then, it can function as an oncomiR or as a tumor suppressor. On the one hand, miR-130b-3p was highly expressed in nephroblastoma, and it was inversely correlated with the expression of phosphatase and tensin homolog (PTEN) protein (Hu and Yan, 2019). Another study showed miR-130b-3p upregulated in neoplastic versus normal prostate tissue, even in mestastatic versus primary sites (Fort et al., 2018). Hirono and others. found miR-130b significantly increased in NSCLC specimens from patients with vascular and lymphatic invasion (Hirono et al., 2019). Altogether these reports agree miR-130b exhibits oncogenic roles. However, another report showed the opposite. Zhao and others observed miR-130b downregulated in 52 pancreatic cancer tissues and 5 cell lines. Moreover, as they overexpressed miR-130b, the proliferation of pancreatic cancer cells was dramatically suppressed both *in vivo* and *in vitro*; similarly, miR-130b remarkably diminished the invasivity of pancreatic cancer cells (Zhao et al., 2013).

Regarding invasion and metastasis driven by EMT, Dong and others found an interesting connection with p53 GOF mutants. These authors carried out an array-based miRNA profiling of p53-null HEC-50 endometrial cells transduced with three mutants, p53R273H, p53R175H, and p53C135Y or empty vector; 23 out 188 miRNAs were expressed above background levels. The authors observed an inverse correlation between miR-130-b and these p53 mutants, plus p53R248Q. In turn, the silencing of the mutant p53R248Q in HEC-1 cells led to the upregulation of miR-130b. To determine whether the endogenous p53 mutant can bind to the promoter of miR-130b, chromatin immunoprecipitation (ChIP) and qPCR analysis on HEC-1 cells was made, demonstrated that miR-130b is a direct target of p53 mutant in endometrial cancer (EC) cells.

The *in silic*o analysis predicted binding of miR-130-b to the 3′UTR of ZEB1 mRNA, and such interaction was further demonstrated in HEC50 and HEC-1 cells resulting in the repression of ZEB1. Given the outstanding capability of this transcription factor to promote invasion and metastasis by inducing EMT (Zhang et al., 2015), the authors provided evidence of the induction of metastatic-associated genes such as SPP1, *MMP2*, and *MMP9* as well as EMT-promoting genes like *ZEB1*, *BMI1*, *CDH2* (N-cadherin) and *VIM* (Vimentin) in the presence of p53 mutants (Dong et al., 2013).

These data demonstrate that p53 GOF mutants downregulate miR-130b expression, which results in activation of *ZEB1*, and its downstream pathway contributes to the induction of EMT and increased EC cell invasion (Dong et al., 2013) (Figure 4B; Table 1).

#### miR-218 and miR-519a

As reported by Garibaldi and colleaagues, the mutant p53R273H downregulated miR-218 and miR-519a in colon cancer cells (Garibaldi et al., 2016a). Moreover, the overexpression of these miRNAs impairing the migratory capability of SW480 cells was determined by wound-healing assays. Interestingly, the presence of these miRNAs was associated with a lower level of *Zeb1* and upregulation of *CDH1* (E-cadherin); the *in silico* analysis indicated one putative binding site for miR-218 and two sites for miR-519a on the 3’ UTR of *ZEB1*. In agreement, another report showed that miR-218 diminished the invasion and metastasis of gastric cancer by suppressing the Robo1 receptor, which activates the slit-Robo1 pathway triggering metastasis *in vivo* and *in vitro* (Tie et al., 2010). Similarly, when miR-218 was restored in glioma cells, cell migration, invasion, and proliferation dramatically reduced, presumably by targeting the stem cell promoting oncogene *BMi1* (Tu et al., 2013).

Regarding miR-519a, its expression was markedly diminished in cancer tissues from the ovary, lung, and kidney, and this was correlated with high levels of the RNA-binding protein HuR. In turn, when miR-519 was overexpressed in human cervical carcinoma xenografts, the tumor size was significantly smaller than controls (Abdelmohsen et al., 2010). Altogether, the evidence strongly suggests that mutants of p53 can promote EMT of tumor cells by inhibiting miR-218 or miR-519a (Figure 2; Table 1).

#### Let-7i

The function of the let7 family members is rather versatile. In the vertebrates, the let-7 family, along with miR-100 and miR-125, form a complex system of developmental regulators (Hertel et al., 2012). Besides, the let-7 miRNAs perform diverse functions such as regulators of cell proliferation, cell cycle, migration, progression, stem cell biology, metabolism, and chemoresistance (Mizuno et al., 2018; Chirshev et al., 2019). Some of the let-7 miRNA family members in the brain have been shown to serve as ligands of Toll-like receptors (TLR7) expressed in the microglia. Interestingly, the resulting signaling induces inflammatory cytokines microglial release that modulates antigen presentation and reduces cell migration. Furthermore, the expression of let-7 miRNAs also inhibited the growth of murine GL261 glioma through microglial TLR7 (Buonfiglioli et al., 2019). In accordance, the let-7 miRNAs have been found altered not only in glioblastomas but in an extensive list of cancers such as breast, ovarian, pancreatic, lung, liver, gastric and oral squamous cell carcinoma, among other types of cancers (for an excellent review see Boyerinas and others. 2010) (Boyerinas et al., 2010).

The p53 mutants can influence the let-7 miRNAs expression as well. Subramanian and others sequenced small RNAs from the p53-null H1299 lung cancer cell line, stably transfected with the hotspot aggressive mutant p53R273H (32). In this approach, they observed 38 miRNAs up- and three downregulated; the tumor suppressor let-7i was abundant in the control cells but significantly down-regulated in H1299 cells expressing p53R273H. Stable knockdown of endogenous mutant p53 in MDA-MB-231 (breast cancer, p53R280K) and DLD1 (colorectal cancer, p53S241F) cell lines released the let-7i repression. After that, by ChIP assays from MDA-MB-231 cells, the authors determined that while mutant p53 inhibits the occupancy of p63 on the let-7i promoter, the mutant p53-p63 complex is bound to the let-7i promoter as well, both mechanisms working together to silence the let-7i expression. Interestingly, the breast cancer MDA-MB-231 and pancreatic cancer MIA-PaCa-2 xenografts showed minor migration and invasion when let-7i was introduced. Then, the authors identified the targets of let-7i by microarray assays and validated them by qRT-PCR; these were mainly oncogenes like *E2F5*, *HMGA1*, *MYC*, and *N-RAS*, and genes related to RNA metabolism like *CPSF1*, *DDX18*, and *LIN28B* (Subramanian et al., 2015).

**FIGURE 5:**
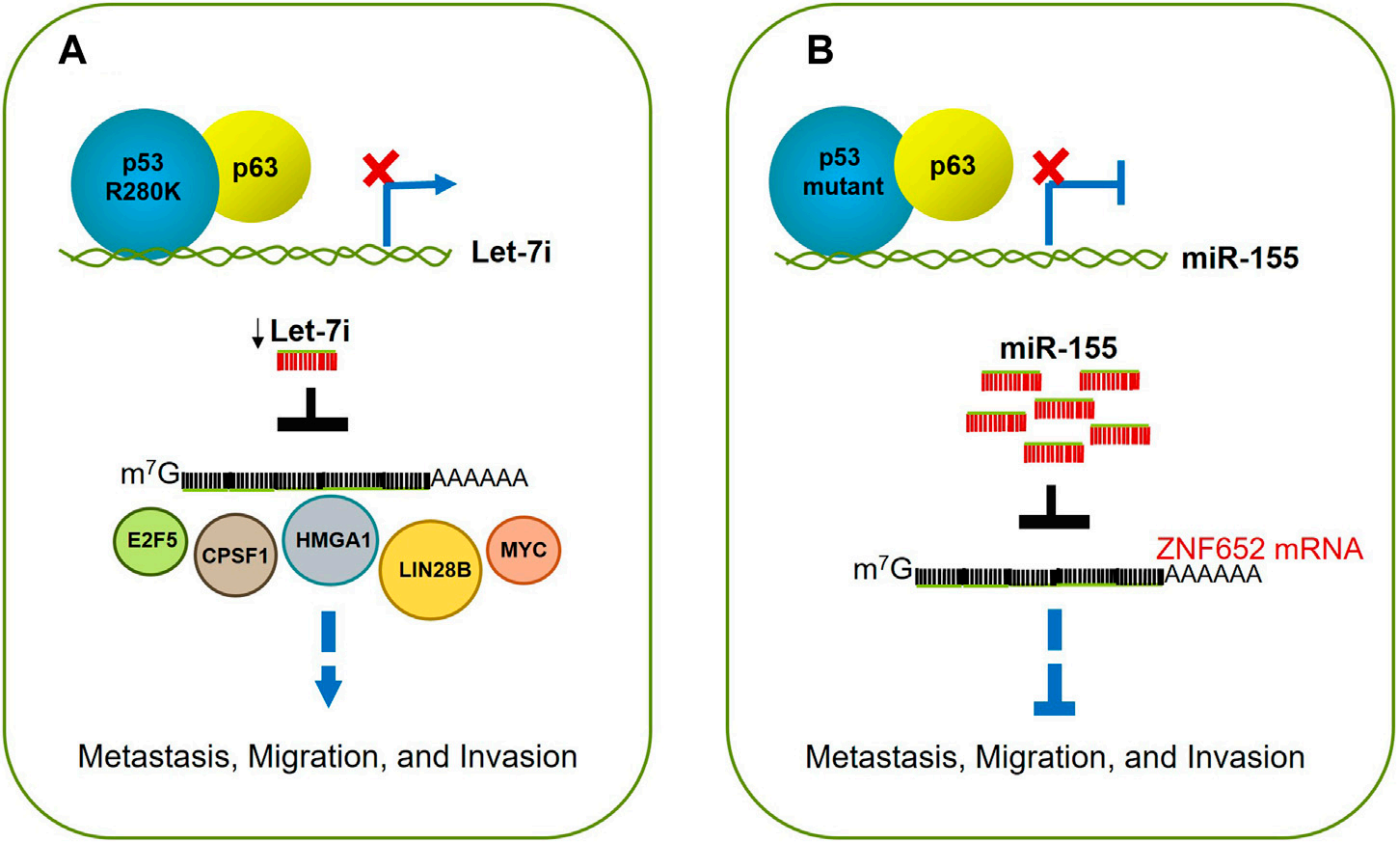
p53 mutants drive migration, invasion, and metastasis. **(A)** p53R280K associates with p63 blocking its occupancy on let-7i promoter; this leads to downregulation of let7i, and increasing levels of its targets like E2F5, CPSF1, HMGA1, LIN28B, MYC, and NRAS. The alteration of this balance fuels cell proliferation, migration, and invasion. **(B)** Mutant p53 (p53R248Q or p53R282W) acquires enhanced invasive and metastatic potential through up-regulation of miR-155. This oncomiR promotes invasion by directly repressing the target transcript ZNF652, which as a consequence, causes the acquisition of an invasive cell phenotype.

The transcendental finding of this work is perhaps the reaffirmation of the concept that the mutants of p53 exert a dominant negative effect over their wild type counterpart and further to the other members of the p53 family, in this case, the p63 protein; the outcome of this interaction, at least in this report, turned out in cellular proliferation, migration, and invasion (Figure 5A; Table 1).

#### miR-155

miR-155 is a master regulator of the immune system. It is expressed mainly in the thymus and spleen and in lesser extension in other tissues (Mashima, 2015). It was originally identified as a gene B-cell integration cluster (bic), considered a proto-oncogene in chickens, then it was realized that miR-155 is expressed from bic exon 2 (Vigorito et al., 2013).

The expression of miR-155 in the immune cells such as macrophages or dendritic cells, leads to cytokines positive regulation. miR-155 can modulate activated myeloid and lymphoid cells transcriptome that influences several biological functions such as inflammation or immunological memory (Vigorito et al., 2013). Regarding cancer, this micro-RNA has shown a protagonist role. miR-155 is correlated with poor prognosis in patients with bladder cancer (Wang and Men, 2015); its expression, along with miR-27a, promoted tumor metastasis and chemoresistance in gastric cancer (Li et al., 2020). Although miR-155 is majorly identified as an oncomiR, its overexpression prevented the cell migration and invasion of HT-29 gastric cancer cells (Liu et al., 2018).

Interestingly, Nielsen and others showed the interconnection between mutants of p53 and miR-155 and their role in promoting cell migration and invasion. Using a scratch-wound assay, they first observed an enhanced ability to migrate the ZR-75-1 and MCF10A epithelial breast cancer cells, both stably transfected with miR-155. Moreover, the expression of miR-155 was tightly associated with the presence of mutants of p53, since the induced expression of either p53R248Q or p53R282W in the p53-null H1299 cells was associated with a dose-dependent increased expression of mature miR-155 levels while knockdown of endogenous mutant p53 in BT-549 (p53R249S) down-regulated miR-155, suggesting that miR-155 is a mutant p53 target in breast cancer cells.

As mentioned before, some mutants of p53 are known to impair the function of p63. By ChIP experiments, the authors demonstrated that in MCF-10A cells, the endogenous p63 was directly recruited to consensus p63-Response Elements, localized within the promoter of the miR-155HG microRNA host gene, in the absence of mutant p53; this finding suggests that p63 represses miR-155 and that this repression is canceled by mutant p53; however, they did not demonstrate a direct association between mutants of p53 and p63 to support this mechanism.

By performing a thorough analysis in the literature, the authors identified 42 candidate genes that drive invasion in breast tumors, downregulated by mutant p53 presumably through miR-155. From this list, four genes (*ZNF652*, *PDCD4*, *TCF12*, and *IL17RB*) were downregulated in tumors with the highest frequency of metastasis-related poor outcomes, being *ZNF652* the best candidate for further investigation since it was previously demonstrated to be downregulated by miR-155. The *ZNF652* gene, encodes for a transcription repressor factor (Kumar et al., 2006; Kumar et al., 2008), which was shown to be directly recruited to gene regulatory elements of *TGFB1*, *TGFB2, TGFBR2, EGFR, SMAD2,* and *VIM* through ChIP analysis in ZR-74-1 cells. Besides, the silencing of *ZNF652* led to the upregulation of these genes, indicating that this gene is indeed required for their repression. To acquire more knowledge about the extent of *ZNF652* influence, the authors performed gene expression profiling in the absence of *ZNF652* or overexpressing miR-155. They observed a correlation between the EMT gene expression program in the absence of *ZNF652* or the presence of miR-155, both converging within the TGF-β signaling pathway (Neilsen et al., 2013). Accordingly, the *ZNF652* levels were lower in malignant invasive breast tumors in comparison with normal breast tissue.

Altogether, these findings support the hypothesis that mutants of p53 can promote invasion and metastasis because of the up-regulation of miR-155. In turn, this oncomiR triggers the EMT program, involving the TGF-β pathway, through the repression of the tumor suppressor *ZNF652* (Neilsen et al., 2013) (Figure 5B; Table 1).

### Vesicular Secretion

The extracellular vesicles are involved in cell-to-cell communication. The cargo can be lipids, proteins, or nucleic acids. In a cancer scenario, the cancer cells-delivered vesicles are uptaken by the tumor microenvironment cells, which can foster the tumor progression, metastasis or drug resistance (Maleki et al., 2021). One of the most overexpressed microRNA in cancer-derived exosomes is the miR-21. It was found that this oncomiR is transferred from cisplatin-resistant oral squamous carcinoma cells through exosomes, and this transference conferred resistance to cisplatin-sensitive cells by targeting *PTEN* and *PCD4* (Liu et al., 2017). Previously, it was observed an association between abundant expression of miR-21 and human metastatic tumors harboring p53 mutations (Bornachea et al., 2012). After that, other researchers linked the overexpression of miR-21 with specific mutations in p53 (R175H and R248Q); however, the overexpression of this oncomiR is just a piece of the landscape since p53 mutants, as described below, can also remodel the extracellular matrix and improve vesicle secretion thus fostering cell migration and drug resistance.

#### miR-30d

It has been recently demonstrated that p53 mutants (R175H, R273H, and R280K) can induce structural changes in the Golgi Apparatus. These changes were mainly related to an increase in the number of *cis*-Golgi elements and a replacement of perinuclear ribbon-like structure by multiple mini-stacks dispersed within the cytoplasm. In addition, the p53 mutant-induced Golgi remodelation increased notably the vesicular trafficking and secretion. The underlying mechanism seems to imply that p53 mutants form a complex with HIF1-alfa that transactivate miR-30d; then, this miRNA represses *AP2A1, DGK2, PPP3CB,* and *VPS26B*, all associated with vesicular trafficking and recycling processes.

This increasing secretion provoked some interesting changes in the extracellular matrix (ECM). By atomic force microscopy, the authors observed that p53 mutants enhance the ECM rigidity; then, after analyzing the content of proteins secreted by mutant p53-knockdown in MDA-MB231 cells, a significant decrease in the levels of fibronectin, laminin V, and laminin B1 was observed. It is not surprising that all these changes led to the alteration in cell migration. When H1299 cells were exposed to a medium collected from MDA-MB231 cells, their migration capability increased more than two-fold. Accordingly, when immunodeficient mice were injected with luciferase-expressing MDA-MB-231 cells defective in the expression of miR-30d, the tumor growth was delayed over a period of 4 weeks, and notably, the lung colonization was dramatically reduced too (Capaci et al., 2020). As expected, the use of the drug PRIMA-1MET, which restores p53 wt function, resulted in the downregulation of miR-30d in MDA-MB231 cells.

Considering that this mechanism involves vesicular secretion, it would be interesting to explore whether the use of drugs that affect this route may impair the p53 mutant-induced vesicle trafficking and exosome secretion. In fact, some drugs like anti-malarial chloroquine and its derivative hydroxychloroquine are widely accepted for their ability to impair autophagy (Mauthe et al., 2018). Yet, their capability to de-acidify lysosomes and Golgi apparatus also preclude the vesicular secretion (Halcrow et al., 2021). Then a combinatorial therapeutic scheme seems to be a good choice in the appropriate context, that is, in the presence of p53 mutants. Altogether, these authors provided interesting evidence that showed how p53 mutants increase the vesicular trafficking and secretion, which favors the generation of a microenvironment permissive for metastatic colonization (Capaci et al., 2020).

Furthermore, the p53 mutants could facilitate the delivery of vesicles charged of oncomiRs through this recently described mechanism. For instance, it was observed that miR-105 can be transferred via exosomes from huvec cells; the resulting impact on the uptaking cells was the loss of *Z O -1* expression and thereby the loss of the epithelial architecture and polarity (Zhou et al., 2014). In accordance, it was observed that the co-culture of macrophages with gastric cancer cells or ovarian cancer cells led to an induction of chemoresistance in both cell lines. The underlying mechanism involved the uptake of macrophage-derived exosomes enriched with miR-223 by the gastric- and ovarian cancer cells (Zhu et al., 2019; Gao et al., 2020b). Since it was not specified the status of p53 in the macrophage THP1 cells, it remains to explore whether p53 mutants improve the exosome secretion. Also, it is unclear if the mutant-p53 inducing overexpression of miR-21 in oral squamous cancer cells is also linked to its vesicular packing and secretion.

## Regulation of miRNA Biogenesis by Mutant p53

So far, the knowledge about the underlying misregulation of miRNAs in cancer, rather than mutations or deletions, seems to be oriented to an imbalance of miRNA levels by impaired maturation at pri- or pre-miRNA processing steps (Calin and Croce, 2006). For an excellent review about miRNA biogenesis in the context of mutants of p53, see the manuscript of Aymone Gurtner and collaborators (Gurtner et al., 2016). For instance, by using the p53 wild-type HCT116 human colon cancer cell line, Suzuki and others determined that p53 wt can enhance the post-transcriptional maturation of several miRNAs with a growth-suppressive function such as miR-143 and miR-16-1 that target K-Ras, and miR-145 which targets CDK6 in response to DNA damage. The authors observed that p53 wt interacts with the Drosha complex through p68/p72 helicases; the carboxy-terminal half of the DNA-binding domain of p53 is the interacting region with p68 or Drosha complex. On the contrary, the p53 mutants R273H, R175H, and C135Y interact in a lesser extension with p68, leading to attenuation of miRNA processing activity (Suzuki et al., 2009).

**FIGURE 6:**
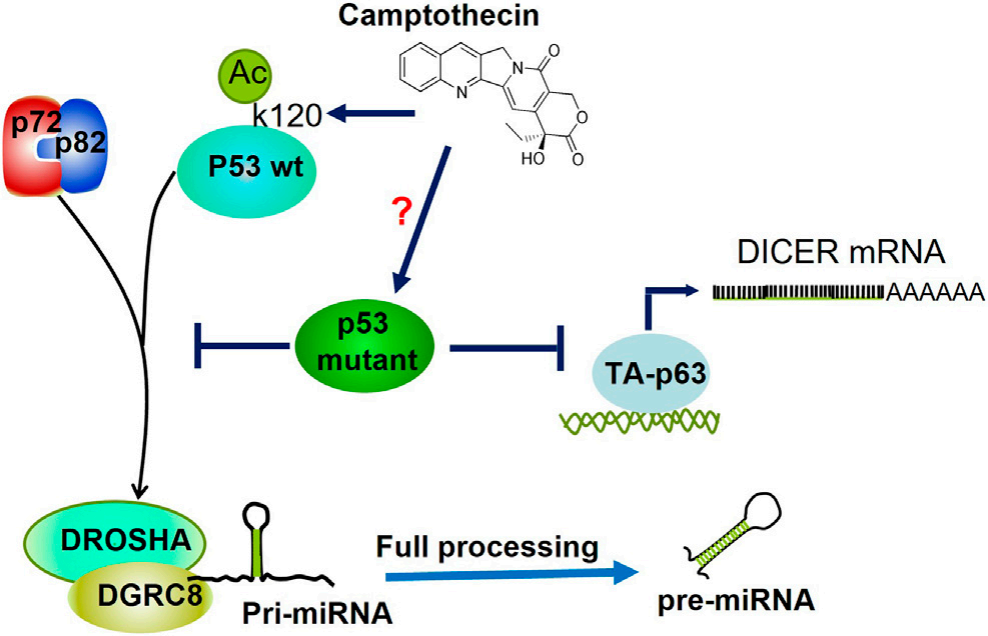
Regulation of miRNAs processing by Mutant p53. Mutants of p53 hinder the association between RNA helicases p72/82 and the microprocessor complex (DROSHA-DGCR8), thereby inhibiting miRNAs' maturation post-transcriptional level. Camptothecin induces acetylation of K120 within the DNA Binding Domain of p53, which augments its association with p72/p82. Whether this drug may revert the poor association of p53 mutants with p72/p82 is not known. p53R273H inhibits the transcription of DICER mRNA through both direct and indirect inhibition of transactivation of TA-p63.

Similarly, Chang and others observed that camptothectin-induced acetylation in lysine 120 (K120) within the DNA-binding domain of p53 promoted its association with the Drosha complex leading to improved processing of miR-203. However, augmenting the maturation of miR-203 blocks Bcl-2, thus inducing the p53-dependent cell death (Chang et al., 2013). Since K120 is not one of the p53 hot spot mutations, its acetylation on p53 mutants via camptothectin would be an interesting approach to observe whether this drug may revert the affinity loss towards Drosha observed in p53 mutants (Figure 6).

Following these findings, Jiang and others reported that mutants of p53 (C135Y, R175H, R248Q, and R273H) promoted EMT in endometrial carcinoma (EC) a similar mechanism. As opposite to p53 wt, the mutants of p53 induced poor processing and maturation of pri-miR-26a-1 since its respective pre-miR-26a-1 mature form was diminished in the presence of the mutants of p53. The lack of maturation resulted from a loss of interaction between the Drosha complex and p68 induced by these mutants. The target of miR-26a is Enhancer of Zeste Homolog 2 (*EZH2*), the catalytic subunit of the Polycomb Repressive Complex that is involved in EMT and is best known to repress a large number of tumor suppressor genes. Stepwise, the authors showed that overexpression of miR-126a reverted the EMT phenotype in HEC-1B cells (Jiang et al., 2015).

As mentioned previously, Garibaldi and others demonstrated that endogenous mutant p53R273H binds to and sequesters RNA heliscases p72/82, thus interfering with its association with Drosha and therefore precluding pri-miRNAs processing and biogenesis. In agreement, they determined that p53-R175H and -R280K can interact with p72/82 through its N-terminal domain. Since mutant p53 does not bind to pri-miRNAs, then p72/82 complex recruitment is pri-miRNA independent. Moreover, the analysis of pri-miRNA, pre-miRNA, and mature miRNA expression shows that mutant p53 downregulates miRNAs at the transcriptional and post-transcriptional levels. Consistent with this, p53wt has an opposite effect on the expression of mutant p53-repressed miRNAs on colon cancer cell lines (Garibaldi et al., 2016b). Besides, Muller and others delineate a clear relationship between mutant p53, p63, and Dicer that might contribute to the metastatic function of mutant p53. They showed that mutant p53 can downregulate Dicer expression through both direct and indirect inhibition of the TAp63-mediated transcriptional activation of Dicer. Consistent with this, the transient expression of mutant p53R273H in H1299 caused a decrease in endogenous Dicer mRNA and protein expression (Muller et al., 2014) (Figure 6).

In an integrative effort, another study involved the use of multi-omics technologies to identify the common targets among five mutants of p53, such as p53R175H and p53R273H, in triple negative breast cancer cell lines (TNBC), the study showed that proteasome machinery is particularly overactivated. The proteasome activity targets for degradation of some tumor suppressors like p21, p27, and *NOXA*; interestingly, it also targets KSRP, which is crucial for the maturation of microRNAs. Even more interesting was the counteracting effect of the co-treatment of carfilzomib (a proteasome inhibitor) and PRIMA-1MET (a mutant p53-inactivating agent) on TNBC cells that resulted in a markedly diminished chemoresistance (Walerych et al., 2016; Di Agostino, 2020).

## Concluding Remarks

For a while, it has been shown the characteristic capabilities of p53 mutants not just for losing their wild type functions but also for acquiring new ones. Among these properties, their ability to modulate both coding and non-coding RNAs is outstanding. The astonishing fact that some miRNAs behave differently according to the cellular context opens a new research path to explore; one possible explanation may come from observations of Vasudevan and others. Strikingly, they observed that microRNAs could make a switch that activates the protein expression depending on the cellular environment instead of silencing a target gene (Vasudevan et al., 2007). Specifically, when cells were under serum starvation, they became quiescent, and in this state, they exhibited upregulation of a reporter gene. The authors determined that AGO-2 (Argonaute) and FXR1 (Fragile X mental retardation-related protein 1) are involved in the miRNA switch only in cells under stress but not in proliferating unstressed cells. This fact could aid in explaining that under certain circumstances or stress conditions, the microRNAs change their role from silencers to translation inducers. In a cancer scenario, where hypoxia or nutrient starvation are common events, it could trigger the converse response of microRNAs. Whether p53 mutants regulate the cellular environment to influence this condition is unknown; for instance, the expression of FXR1 is found upregulated in colorectal cancer (Jin et al., 2016). Then, it would be interesting to search if p53 mutants can upregulate this oncogene. Another plausible explanation for this duality may be due to the diverse and wide number of targets that one miRNA can have. In other words, one miRNA can target several oncogenic mRNAs and several tumor suppressive mRNAs simultaneously, so the balance of both may dictaminate the final cell fate. For instance, miR-125 can silence a plethora of mRNAs whose function is opposed to each other. For example, it targets antiapoptotic factors (BCL2, BCL2L2, MUC1), proapoptotic factors (TP53, BAK1, PUMA), metastatic factors (MMP1, VEGFA, ERBB2/3) among others (Sun et al., 2013). Hence, miR-125 have shown diverse facets, as an oncomiR in most hematologic malignancies and as a tumor suppressor in solid tumors (Svoronos et al., 2016). Another contrasting behavior is presented by miR-155. As we mentioned above in the “EMT section”, this miRNA is associated with mutant p53 expression and also is correlated with EMT and migration in breast cancer cells (Figure 5B); however, under ionizing radiotherapy miR-155 favour an apoptotic outcome by targeting RAD51 (Gasparini et al., 2014), which is a critical factor for the DNA repair thorugh homologous recombination. The more affected the DNA repair machinery is, the more susceptible the cell is under DNA damage, which turns out in better overall survival**.** Given that miRNAs regulate approximately 60% of the global expression of genes, it is not surprising the growing number of reports that associate a determined miRNA profile with a specific disease. The mutants of p53 can impinge the maturation of miRNAs, resulting in the imbalance of miRNAs levels in some types of cancers, thus producing a specific miRNAs expression profile. In fact, Zhang and others in 2016 identified a miRNA signature that correlated with poor cancer outcomes. Using a microarray method, they observed the miRNAs either up-or downregulated in response to the expression of the p53mutant R282W in H1299 cells. Once analyzed by the non-negative matrix factorization model and Kaplan-Meier test, these data revealed a mutant-p53 signature identifying cancer subgroups with significantly different outcomes. These observations were reproducible for liver, breast and gastric cancer (Zhang et al., 2016). In another report, there were examined 121 patients with HNSCC to explore the status of p53. From these cohorts of patients, it was identified a signature of 12 miRNAs showed an association with shorter recurrence-free survival among those that harbored p53 mutations (58%) (Ganci et al., 2013; Valenti et al., 2019).

In addition, several studies show that patients with tumors carrying p53 mutations have shorter survival, worse prognosis and poorer response to conventional anti-cancer treatments. In order to tackle this, scientists are developing several drugs targeting the mutations already discussed in this issue; then, it would be very interesting to assess whether these drugs may revert the altered expression of miRNAs provoked by mutants of p53. Besides, the recently described modes of action of p53 mutants also open new treatment alternatives, specifically by tackling the secretory pathway. In this regard, chloroquine, hydroxychloroquine and Ivermectin are promise drugs that have a great anti-cancer potential. With this knowledge, this therapy hopefully could be more focused among patients harboring p53 mutations.

There remain some open questions, for instance, given that p53 mutants impair the function of factors that regulate the overall maturation machinery of microRNAs, which is perhaps the reason that p53 mutants induce an overall dowregulation of microRNAs, then we wonder ¿how do the mutants of p53 do for up-regulating specific microRNAs? or ¿how do they do to down-regulate some microRNAs in a quite larger extension than others?

In summary, the full comprehension of the molecular events underlying gain of function of mutant p53 proteins is essential for improving our understanding of the acquisition of aggressive traits of carcinoma cells, such as invasion and metastasis. It thus may help us to propose new target therapies.

## Glossary

WT: wild-type
UTR: untranslated region
p53: tumor suppressor protein
TP53: tumor protein 53 gene
miRNA: short non-coding RNAs
RISC: silencing complex
AGO2: argonaute 2
TRBP: TAR RNA binding protein
PACT: double strand dsRNA-binding protein
ChIP: chromatin immunoprecipitation
EGFR: epidermal growth factor receptor
ERK1/2: mitogen-activated protein kinases
ARPP21: cAMP-regulated phosphoprotein 21
DOXO: doxorubicin
CDDP: cisplatinum
5-FU: 5-fluorouracil
E2F5: transcription factor 5
p21: cyclin-dependent kinase inhibitor
ZEB1: zinc finger E-box binding homeobox 1
STMN1: stathmin 1
Pyk2: pyruvate kinase
PARP: poly-(ADP-ribose) polymerase
GOF: gain-of-functions of mutant p53
IP: propidium iodide
MMP-2: matrix metallopeptidase 2
MMP-9: matrix metallopeptidase 9
BMI1: polycomb ring finger oncogene
HuR: RNA binding protein
EV: empty vector
CPSF1: cleavage and polyadenylation specific factor
HMGA1: high mobility group AT-hook 1
LIN28B: lin-28 homolog B
MYC: v-myc avian myelocytomatosis viral oncogene homolog
NRAS: neuroblastoma RAS viral oncogene homolog
DDX18: DEAD-box helicase 18
DDX17: DEAD-box helicase 17
DDX5: DEAD-box helicase 5
TGF-β: transforming growth factor
p63: tumor suppressor protein
p63-RE: p63 response element
ZNF652: zinc finger protein 652
PDCD4: programmed cell death 4
TCF12: transcription factor 12
IL17RB: interleukin 17 receptor B
SMAD2: SMAD family member 2
DGCR8: DiGeorge critical region 8
Cdk1: cyclin dependent kinase 1
MAPK: mitogen activated kinase-like protein
EGF: epidermal growth factor
Robo1: roundabout 1
TGFB1: transforming growth factor beta 1
TGFB2: transforming growth factor beta 2
TGFBR2: transforming growth factor beta receptor 2
TAp63: p63 isoform.
